# Modeling the drivers of mumps incidence in China: a spatial multi-method analysis

**DOI:** 10.3389/fpubh.2026.1805283

**Published:** 2026-05-19

**Authors:** Ke Hu, Xingjin Yang, Shuiping Ou, Chaojie Li, Xing Zhang, Di Xiao, Mingyang Yu

**Affiliations:** 1 Xiamen Haicang Hospital, Xiamen, Fujian, China; 2 QianDongNanZhou Center for Disease Control and Prevention, QianDongNanZhou, Guizhou, China; 3 Honwing Pharma (Guizhou) Company Limited, QianDongNanZhou, Guizhou, China; 4 Xingtai Center for Disease Control and Prevention, Xingtai, Hebei, China; 5 Nanjing Lishui Dongping Street Health Center, Nanjing, Jiangsu, China; 6 Community Health Service Center of Jiuxian Tongliang District, Chongqing, China; 7 Fuwai Central China Cardiovascular Hospital, Zhengzhou, Henan, China

**Keywords:** geographically weighted regression, influencing factors, multiple linear regression, multiscale geographically weighted regression, mumps incidence, spatial heterogeneity, spatial lag model

## Abstract

**Introduction:**

Mumps poses a considerable public health burden in China and exhibits significant spatial heterogeneity. Systematically exploring its multi-scale driving factors is crucial for informing targeted public health interventions. However, previous research often relied on global models or single-scale local models, limiting the precise identification of region-specific determinants.

**Methods:**

Using provincial-level data from China in 2020 (the most recent complete year with publicly available multi-source data at the time of analysis), this study employed spatial autocorrelation analysis to detect clustering patterns of mumps incidence. A multi-model analytical framework was then constructed by integrating multiple linear regression (MLR), spatial lag model (SLM), geographically weighted regression (GWR), and multiscale geographically weighted regression (MGWR). While MLR, SLM, GWR, and MGWR are individually established methods, their integrated application within a single analytical framework - coupled with systematic model comparison and residual spatial diagnostics - represents a methodological advancement that enables explicit evaluation of the added value of capturing spatial heterogeneity and scale-dependent effects. This comprehensive approach enabled systematic elucidation of spatial differentiation patterns and scale-dependent driving mechanisms of mumps incidence, overcoming the limitations of traditional global models. The analysis incorporated multidimensional data encompassing socioeconomic factors, education level, healthcare resources, population structure, and environmental factors.

**Results:**

Mumps incidence exhibited a west-high/east-low gradient distribution with significant spatial autocorrelation (*Moran’s I* = 0.399, *p* < 0.001), characterized by high-high and low-low clustering. Model comparisons showed that while MGWR achieved the highest explanatory power (*R^2^* = 0.769; *AIC* = 62.409) and eliminated residual spatial autocorrelation (Moran’s I = −0.0476, *p* = 0.90), its variable-specific bandwidths all exceeded 7,500 km - beyond China’s maximum inter-provincial distance - causing it to degenerate into a global model. In contrast, GWR (*R^2^* = 0.738; *AIC* = 65.918) employed a unified optimal bandwidth of approximately 1,433 km, effectively capturing local spatial heterogeneity. GWR results revealed notable spatial heterogeneity in influencing factors: the negative effect of GDP per capita was strongest in the southwest; years of education showed a pronounced positive effect only in Fujian and Guangdong; the positive association with general practitioner density was most evident in the southeast; PM_2.5_ exhibited a strong negative association in the west; and the child dependency ratio’s positive effect was most prominent in the northeast.

**Conclusion:**

This study quantifies the spatially varying effects of key drivers on mumps incidence across China. Although MGWR offers theoretically advantageous, its practical utility is constrained by limited sample size, making GWR a more robust choice for capturing spatial heterogeneity in small-sample settings. The findings provide a nuanced, scientific basis for developing regionally differentiated prevention and control strategies tailored to local epidemiological profiles.

## 1 Introduction

Mumps poses a considerable public health burden in China, ranking second among Class C infectious diseases. The epidemic demonstrates significant spatial heterogeneity and clustered distribution patterns. Epidemiological surveillance data indicate a national average annual incidence rate of 21.44 per 100,000 population, exhibiting distinct spatiotemporal aggregation characteristics (1). Southern provinces including Hunan, Hubei, Guangdong, and Guangxi, along with certain central and western regions, constitute the primary high-incidence areas (1–3). Spatial autocorrelation analysis further reveals persistent hotspot clusters in central-western and coastal regions of Shandong province (4). Notably, the epidemic distribution displays significant spatial spillover effects, where neighboring regions’ socioeconomic and environmental factors may influence local transmission risks through population mobility or shared environmental pathways (2, 5). This spatial non-stationarity suggests the necessity for in-depth investigation into the underlying mechanisms of mumps incidence. This pronounced spatial heterogeneity and evidence of spillover effects underscore the critical need to move beyond traditional global analyses and systematically investigate the localized, multi-scale driving mechanisms underlying mumps incidence.

Existing research confirms that mumps transmission is jointly regulated by multiple systemic factors. Regional disparities in GDP per capita indirectly affected epidemic distribution through variations in vaccine accessibility and healthcare resource allocation efficiency (2, 6, 7). Areas with lower education levels showed reduced compliance with preventive measures due to inadequate health literacy (8), while insufficient density of general practitioners compromised early case detection and management capacity (9). PM_2.5_ exposure demonstrated a dose–response relationship with incidence rates, potentially by impairing respiratory mucosal immunity and increasing susceptibility (10, 11). Demographic characteristics also played a crucial role, as regions where children aged 5–9 accounted for over 40% of the population were more prone to clustered outbreaks (12, 13). Additionally, seasonal variations in meteorological parameters such as temperature and humidity influenced transmission dynamics by modulating viral survival rates (14, 15).

However, previous studies have predominantly employed global regression models that assume constant relationships across space, or single-scale local models that fail to account for the possibility that different drivers may operate at different spatial scales. A systematic, side-by-side comparison of non-spatial, global spatial, and multi-scale local spatial regression models has not been previously conducted for mumps incidence in China, representing a critical methodological gap that limits the precise identification of region-specific drivers and the development of targeted interventions. This methodological gap limits the precise identification of region-specific determinants and hinders the development of targeted interventions.

To address the spatial non-stationarity of influencing factors, this study established a multi-model collaborative analytical framework. Multiple linear regression (MLR) can identify globally significant variables but fails to account for spatial dependence (16). Global spatial regression models, including the spatial lag model (SLM) and spatial error model (SEM), effectively capture spatial dependence by accounting for spatial lag effects and error autocorrelation, respectively (17, 18). The geographically weighted regression (GWR) model reveals local patterns through spatially varying coefficients, but assumes all variables operate at the same spatial scale (19, 20). In contrast, the multiscale geographically weighted regression (MGWR) model overcomes fixed bandwidth limitations by allowing each variable to autonomously optimize its spatial scale, enabling precise identification of differential spatial patterns among variables (21, 22).

The primary innovation of this study lies in the explicit, sequential integration of MLR, SLM, GWR, and MGWR within a unified analytical framework, accompanied by systematic model comparison and residual diagnostics to quantify the added value of accounting for spatial effects and heterogeneity. This approach elucidates the spatial differentiation patterns of mumps incidence and, for the first time, identifies determinants with geographically varying effects - specifically, how the direction and magnitude of each association differ across regions. While MGWR has been applied to other infectious diseases, its use for mumps remains limited, particularly regarding systematic comparison with global and single-scale local models. Addressing this gap is critical, as interpreting how socioeconomic, environmental, and demographic factors operate across space directly informs regionally tailored interventions. Thus, this study aims to provide a nuanced scientific basis for differentiated prevention and control strategies while advancing the application of comparative spatial epidemiological methods. By overcoming the limitations of traditional global models that assume constant relationships, the research outcomes offer decision-making support for targeted measures and demonstrate a rigorous framework applicable to other infectious diseases.

## 2 Methods

**Figure 1:**
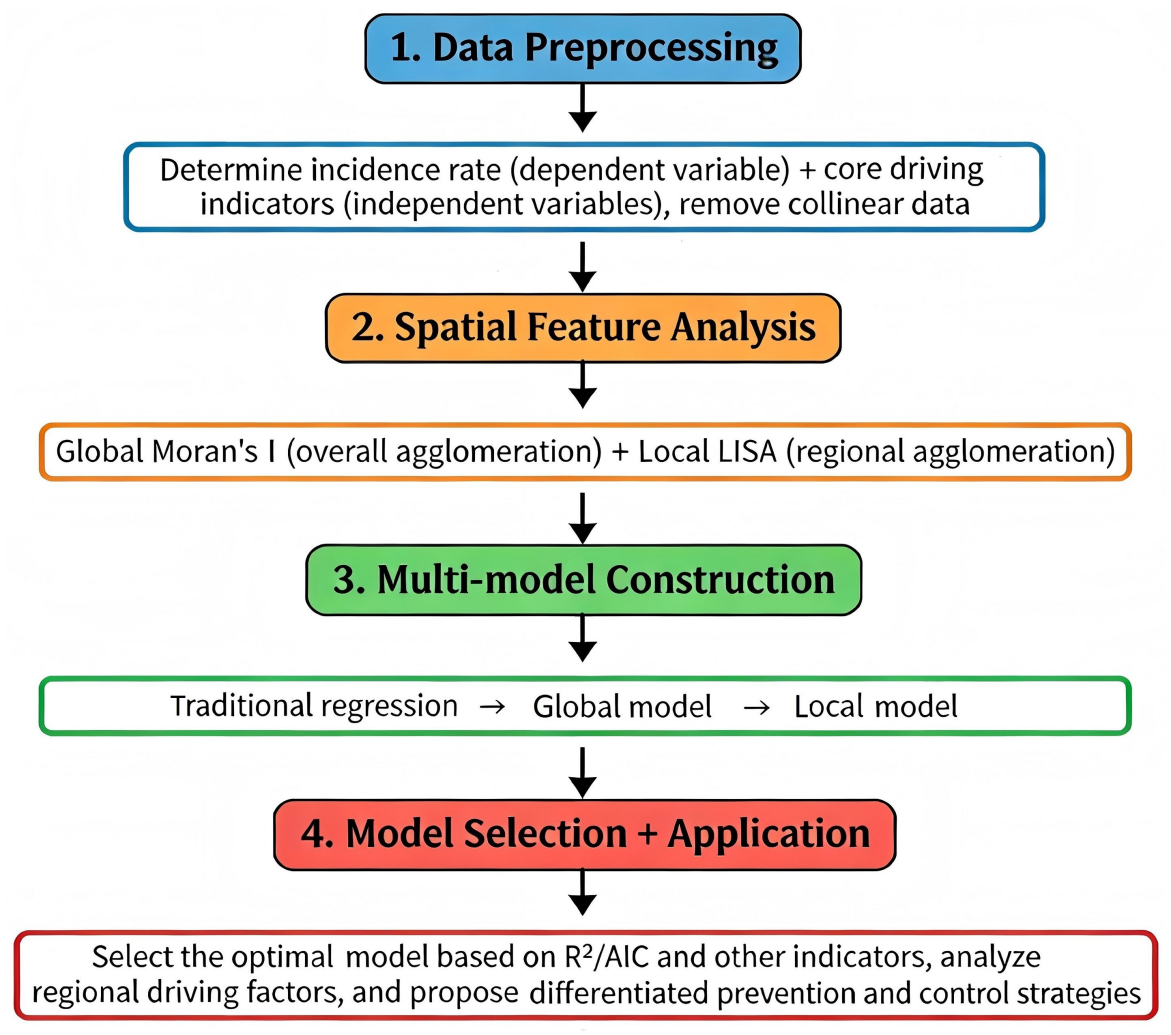
Four-stage analytical framework for spatial modeling.

A four-stage analytical framework was employed in this study, progressing from data preprocessing through spatial feature analysis, multi-model construction, and finally model selection for policy translation (Figure 1).

### 2.1 Data

**Figure 2:**
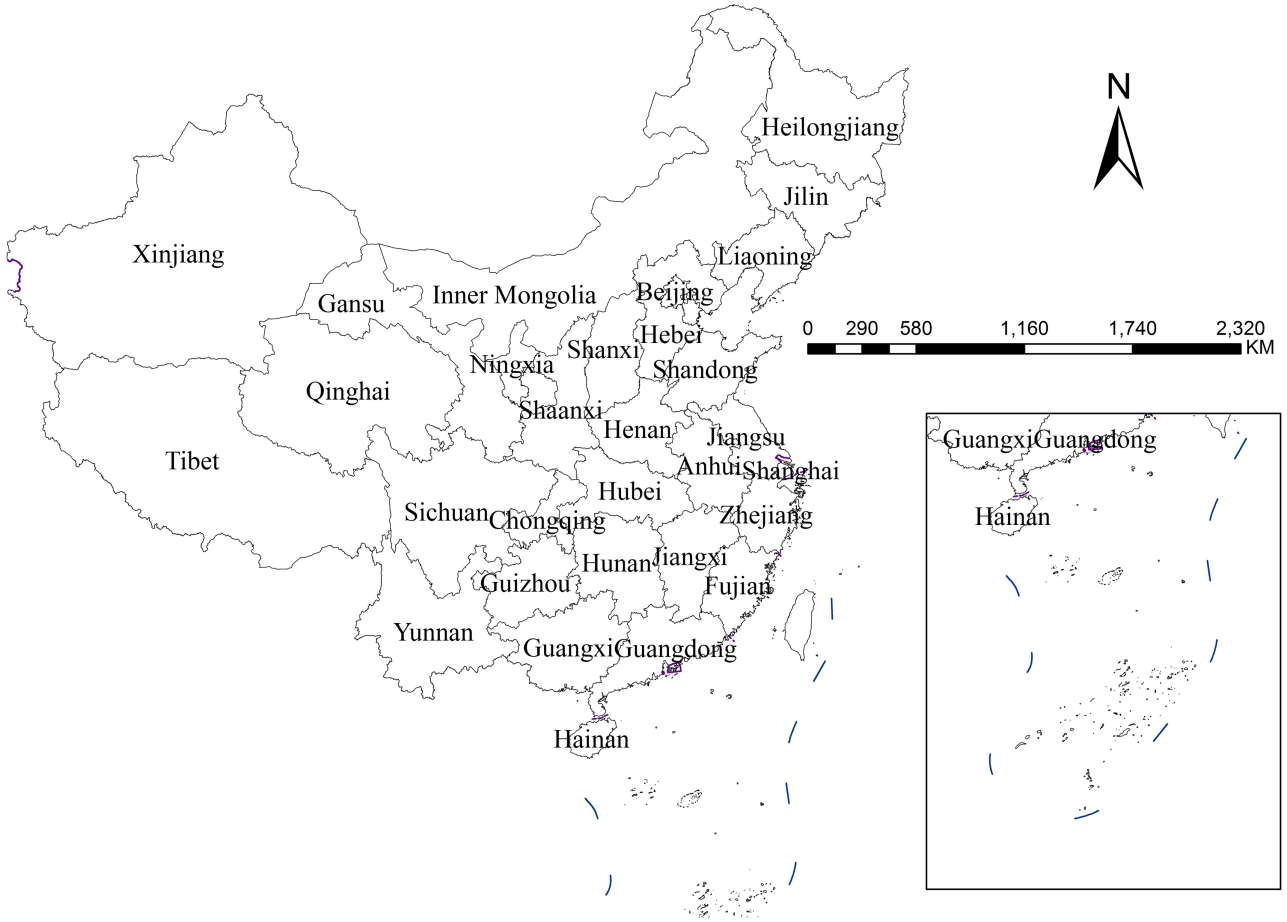
Map of 31 provincial-level administrative divisions in Chinese mainland [the inset in the lower right corner shows the South Sea Islands of China (Map Approval No.: GS (2024) 0650)].

Drawing upon the theoretical framework set forth in the introduction and taking into account methodological considerations regarding data availability, this study ultimately identified seven key indicators covering multiple dimensions including economic development, education level, population structure, environment condition, healthcare resources, consumption level, and urban–rural structure. The analysis used provincial-level administrative regions as the basic units, encompassing 31 provinces, autonomous regions, and municipalities in mainland China, as specifically shown in Figure 2. All data used in this study are annual secondary data for the year 2020, obtained from publicly accessible official sources. The year 2020 was selected as the study period because it represented the most recent year for which provincial-level mumps incidence data were publicly available at the time of analysis.

The mumps incidence data in 2020 were sourced from the Public Health Science Data Center of the Chinese Center for Disease Control and Prevention.^1^ PM_2.5_ concentration data in 2020 were obtained from provincial environmental status bulletins, and other explanatory variables in 2020 were all collected from officially published data in the China Statistical Yearbook (See Table 1 for details). Prior to model estimation, all variables were standardized to have a mean of zero and a standard deviation of one (Z-score normalization). This standardization was necessary for two reasons. First, it eliminates the influence of different measurement units and scales among predictors (e.g., per capita GDP in ten thousand CNY vs. PM_2.5_ in μg/m^3^), ensuring that bandwidth selection and coefficient estimation in GWR and MGWR are not unduly influenced by variable magnitude. Second, it allows direct comparison of the relative importance of different drivers across models. Standardization was applied before any spatial weighting or model fitting, and all reported coefficients in the results correspond to standardized variables.

**Table 1:** Key factors selected for analysis.

Categories	Factors	Data sources
Economic development	GDP per capita	China Statistical Yearbook
Education level	Years of education	
Healthcare resources	Number of general practitioners per 10,000 population	
Population structure	Child dependency ratio	
Consumption level	Household consumption expenditure per capita	
Urban–Rural structure	Urbanization rate	
Environment condition	PM_2.5_	Provincial environmental status bulletins

### 2.2 Descriptive spatial analysis

Geographic visualization methods were employed to create spatial distribution maps of mumps incidence rates across China’s provincial-level administrative regions, providing an intuitive display of disease characteristics in different areas.

### 2.3 Spatial autocorrelation analysis

The spatial distribution patterns of incidence data were systematically evaluated using spatial autocorrelation tests at two levels. A row-standardized first-order queen contiguity spatial weight matrix was constructed from provincial boundaries, where a weight of 1 was assigned to provinces sharing a common vertex and 0 otherwise. This matrix was employed for global Moran’s I and Local Moran’s Index (LISA) calculations.

#### 2.3.1 Global spatial autocorrelation analysis

Moran’s I index was employed to quantitatively measure the spatial clustering degree of mumps incidence nationwide and determine whether significant spatial dependence characteristics exist. The mathematical expression is as follows (Equation 1) (23):

$$I=\frac{n\sum_{i=1}^{n}\sum_{j=1}^{n}W_{\mathrm{ij}}(x_{i}-\bar{x})(x_{j}-\bar{x})}{\sum_{i=1}^{n}\sum_{j=1}^{n}W_{\mathrm{ij}}\sum_{i=1}^{n}{\left(x_{i}-\bar{x}\right)}^{2}}$$ (1)

The *Moran’s I* index ranges from −1 to 1, where *n* represents the sample size, *W*_ij_ denotes the elements of the spatial weight matrix, *x*_i_ and *x*_j_ are observed values, and $\bar{x}$ is the mean value.

Values between 0 and 1 indicate positive spatial autocorrelation, reflecting a clustered distribution of similar values across space. Values between −1 and 0 indicate negative spatial autocorrelation, demonstrating a dispersed pattern with adjacent high and low values. When the index value approximates 0, it suggests no significant spatial association and follows a random distribution pattern. All analytical results require statistical significance verification through Z-test to ensure their reliability (24).

#### 2.3.2 Local Moran’s index (LISA)

The Local Indicators of Spatial Association (LISA) were employed to identify and examine local spatial correlation patterns among spatial units within the study area. The variable definition system remains consistent with the global *Moran’s I* index.

The computational formula is expressed as Equation 2 (25):

$$I_{i}=\frac{n(x_{i}-\bar{x})\sum_{j=1}^{n}w_{j}(n_{j}-\bar{n})}{\sum_{j=1}^{n}{\left(x_{j}-\bar{x}\right)}^{2}}$$ (2)

The method effectively identifies four characteristic spatial association patterns: High-High (HH) clusters representing high-incidence areas surrounded by other high-incidence regions; Low-Low (LL) clusters indicating low-incidence areas adjacent to other low-incidence regions; High-Low (HL) outliers showing high-incidence areas encircled by low-incidence zones; and Low-High (LH) outliers featuring low-incidence areas surrounded by high-incidence regions. All analytical results underwent significance testing and were visually presented using spatial visualization techniques to demonstrate the spatial heterogeneity characteristics of mumps incidence across China (26).

### 2.4 Multiple linear regression (MLR)

This study employed a MLR model to analyze the influencing factors of mumps incidence, with the basic formulation expressed as Equation 3 (27):

$$Y=\beta_{0}+\beta_{1}X_{1}+\beta_{2}X_{2}+\ldots +\beta_{n}X_{n}+\varepsilon \;$$ (3)

Where *Y* represents the dependent variable (mumps incidence rate), *X*_1_ to *X*_n_ denote independent variables of various influencing factors, *β*_0_ is the intercept term, *β*_1_ to *β*_n_ correspond to regression coefficients for respective independent variables, and *ε* is the random error term following a normal distribution N(0,*σ*^2^).

For model evaluation, the coefficient of determination (*R^2^*) was used to measure explanatory power, while the Akaike Information Criterion (*AIC*) balanced model complexity against goodness-of-fit. Multicollinearity was diagnosed using Variance Inflation Factor (VIF), with VIF values exceeding 5 indicating significant multicollinearity issues (28).

### 2.5 Global spatial regression models

The global spatial regression models employed the same first-order queen contiguity spatial weight matrix as used in the spatial autocorrelation analysis.

#### 2.5.1 Spatial error model (SEM)

SEM represents an improved approach addressing spatial autocorrelation in regression residuals. The model specification consists of two components (Equations 4 and 5) (29):

$$Y=X_{\beta }+u$$ (4)

$$u=\lambda W_{u}+\varepsilon$$ (5)

Where *Y* is the vector of the dependent variable (mumps incidence), *X* is the matrix of explanatory variables, *β* is the vector of regression coefficients, and *u* is the residual term. In the error structure, *λ* represents the spatial error coefficient, *W*_u_ denotes the spatially lagged error term, and *ε* is the independent and identically distributed error term following a normal distribution N(0,*σ*^2^).

By incorporating this spatial dependence structure in error terms, the SEM effectively corrects parameter estimation biases that may arise from ignoring spatial autocorrelation in conventional regression analysis (30).

#### 2.5.2 Spatial lag model (SLM)

The SLM accounts for spatial interaction effects between neighboring regions by incorporating a spatially lagged dependent variable. Its mathematical formulation is expressed as (Equation 6) (31):

$$Y=\rho W_{Y}+X_{\beta }+\varepsilon$$ (6)

Where *ρ* represents the spatial autoregressive coefficient quantifying the strength of spatial dependence, and *W*_Y_ reflects the spatial lag effect of the dependent variable, measuring the influence degree from surrounding areas on the explained region. *X* denotes the matrix of explanatory variables, *β* is the vector of regression coefficients to be estimated, and *ε* is the independent and identically distributed random error term. By incorporating this spatially lagged structure, the SLM effectively corrects for potential estimation biases arising from ignored spatial dependence, thereby providing a more accurate delineation of the relationships between influencing factors and disease incidence.

### 2.6 Local spatial regression models

#### 2.6.1 Geographically weighted regression (GWR)

GWR model represents an advanced local spatial regression technique that overcomes the limitation of spatially invariant parameters in traditional global regression models, effectively capturing the spatial non-stationarity characteristics of variable relationships. The model incorporates spatial location parameters, allowing regression coefficients to dynamically adjust according to geographic coordinates. Its basic formulation is expressed as (Equation 7) (25):

$$Y_{i}=\beta_{0}(u_{i},v_{i})+\sum_{k=1}^{n}\beta_{k}(u_{i},v_{i})x_{\mathrm{ik}}+\varepsilon_{i}$$ (7)

Where (*u*_i_,*v*_i_) denotes the spatial coordinates of the *i*_th_ sample point, and *β*_k_(*u*_i_,*v*_i_) represents the spatially varying regression coefficients for the *k*_th_ explanatory variable at location *i*. In model implementation, GWR constructs spatial weight matrices using distance-decay methods based on Gaussian kernel functions, with parameter estimation performed through weighted least squares. A fixed bandwidth was employed, meaning that the spatial weight for each location is based on a constant distance threshold across the study area. The optimal bandwidth parameter is typically determined using optimization methods such as the corrected Akaike Information Criterion (*AIC_c_*), which serves as the primary criterion for selecting the bandwidth that best balances model goodness-of-fit and complexity. Compared with conventional regression methods, the GWR model’s advantage lies in its ability to more accurately characterize the spatial heterogeneity patterns of explanatory variables’ effects on the dependent variable, providing a more refined analytical tool for spatial data.

#### 2.6.2 Multiscale geographically weighted regression (MGWR)

MGWR, an extension of GWR, enhances spatial modeling by utilizing bandwidth parameters that vary by explanatory variable, thereby better characterizing their differing spatial scales of influence. The MGWR model can be expressed as (Equation 8) (29):

$$Y_{i}=\beta_{0}(u_{i},v_{i},bw_{0})+\sum_{k=1}^{n}\beta_{k}(u_{i},v_{i},bw_{k})x_{\mathrm{ik}}+\varepsilon_{i}\;$$ (8)

In MGWR, each variable *k* has its own bandwidth parameter (*bw*_k_). The model uses a back-fitting algorithm to fine-tune these bandwidths iteratively, with the best combination chosen based on the *AIC_c_* criterion. This study adopts the fixed Gaussian kernel function as the basic form of the spatial weight function (32). Compared with traditional GWR models, MGWR demonstrates three core advantages: first, variable-specific bandwidth settings effectively avoid parameter estimation biases caused by single bandwidth; second, it accurately identifies differential characteristics in the spatial influence ranges of different explanatory variables; third, it significantly enhances the model’s analytical precision for complex spatial dependence relationships, providing a more reliable methodological tool for spatial heterogeneity analysis.

### 2.7 Software

This study employed multiple specialized spatial analysis software packages for data processing and model construction:

ArcGIS 10.2 was utilized for explanatory variable discretization, spatial autocorrelation analysis, multicollinearity testing, and spatial visualization of research results.

GeoDa 1.22 was applied to construct and estimate parameters for traditional multiple linear regression models and spatial econometric models (including SLM and SEM).

MGWR 2.2 software was specifically used to perform GWR and MGWR analyses.

All base geographic reference data were obtained from the National Geographic Information Public Service Platform (Map Approval Number: GS (2024) 0650). For all statistical inference procedures, two-tailed tests were conducted with the significance threshold uniformly set at *α* = 0.05.

## 3 Results

### 3.1 Descriptive and spatial autocorrelation analysis results

**Figure 3:**
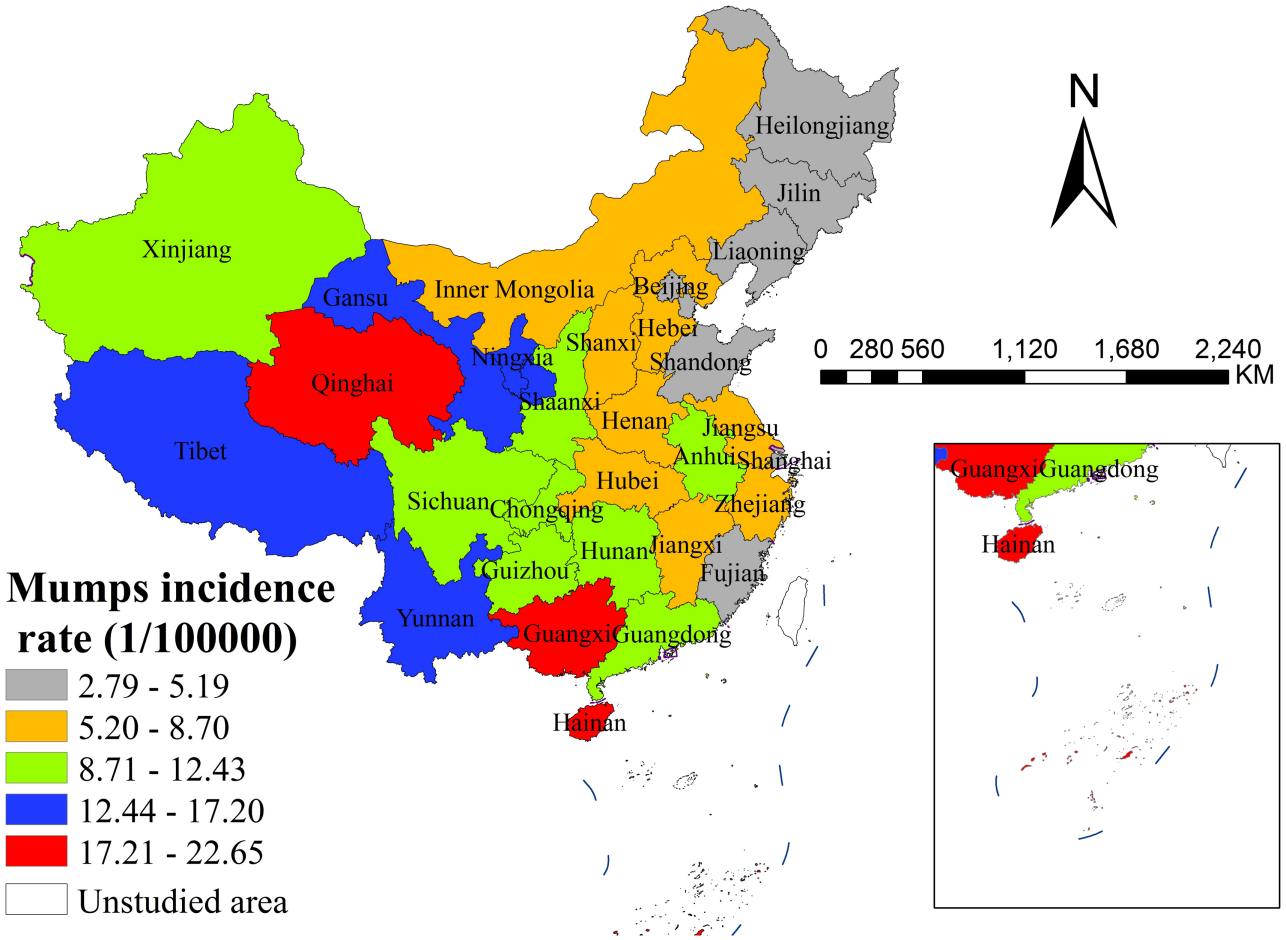
The spatial distribution of mumps incidence in China (2020).

**Figure 4:**
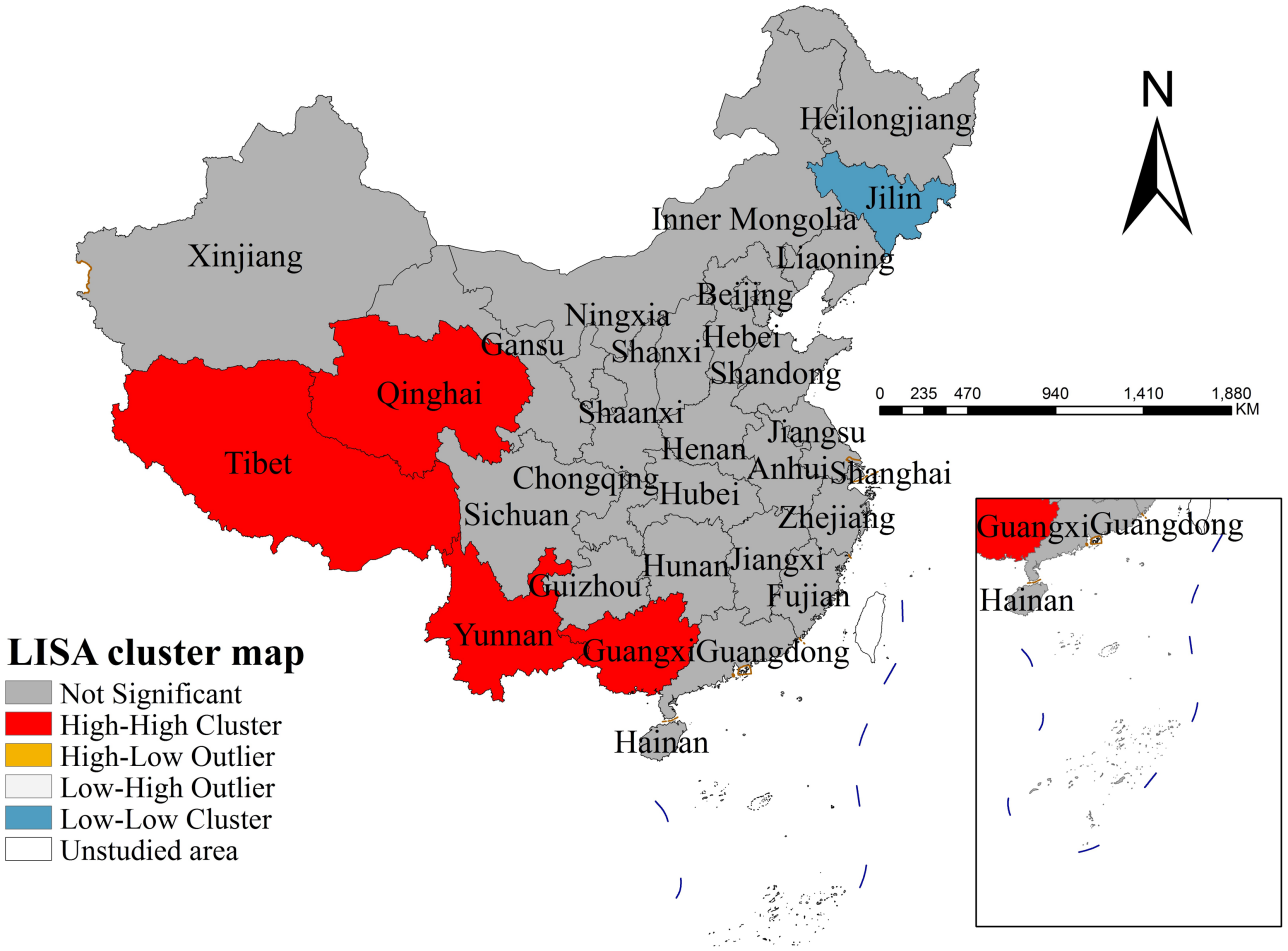
LISA cluster map of mumps incidence (2020).

The results of descriptive and spatial autocorrelation analysis revealed significant geographical disparities in mumps incidence across China in 2020. As shown in Figure 3, the spatial distribution exhibited distinct east–west gradient characteristics, with Qinghai Province reporting the highest national incidence rate (22.65 per 100,000 population) while Heilongjiang Province showed the lowest level (2.79 per 100,000 population). Global spatial autocorrelation analysis confirmed the presence of significant spatial dependence in disease transmission, as evidenced by a Moran’s I index of 0.399 (*p* < 0.001). Further local spatial analysis through LISA cluster mapping (Figure 4) identified concentrated high-high clusters primarily in western provinces including Qinghai, Tibet, Yunnan and Guangxi, contrasting with predominant low-low clusters in Jilin province. These spatial clustering patterns demonstrate substantial geographical heterogeneity in mumps epidemiology across China, highlighting the need for regionally differentiated prevention and control strategies that account for these spatial variations.

### 3.2 Results of MLR and spatial diagnostics

The preliminary analysis identified multicollinearity issues through Variance Inflation Factor (VIF) testing, with household consumption expenditure per capita (VIF = 14.245) and urbanization rate (VIF = 11.166) showing strong collinearity with other explanatory variables, as their VIF values both exceeded the threshold of 5. After removing these two variables, all remaining predictors exhibited VIF values below 5 (Table 2). The initial MLR results indicated that neither years of education nor number of general practitioners per 10,000 population demonstrated statistically significant effects on mumps incidence (Table 2).

**Table 2:** Results of the SLM and MLR.

Factors	SLM		MLR		VIF
	Coefficient	*p*-value	Coefficient	*p*-value	
Intercept	0.017	0.868	−0.000	1.000	/
GDP per capita	−0.559	0.004	−0.521	0.041	3.451
Years of education	0.398	0.073	0.342	0.222	4.411
Number of general practitioners per 10,000 population	0.422	0.009	0.369	0.073	2.288
PM_2.5_	−0.513	0.000	−0.555	0.003	1.612
Child dependency ratio	0.517	0.002	0.634	0.004	2.306
Spatial dependence diagnostics	SLM*: ρ* = 0.440 *p* = 0.008		LM lag: 4.316 (*p* = 0.038) LM error: 1.921 (*p* = 0.166)		/

Spatial diagnostics revealed significant residual spatial autocorrelation (*p* = 0.00907), suggesting the presence of spatial dependence unaccounted for in the conventional regression model. Lagrange Multiplier tests further guided model selection, with the significant Lagrange Multiplier lag test (LM lag = 4.316, *p* = 0.038) and non-significant Lagrange Multiplier error test (LM error = 1.921, *p* = 0.166) collectively suggesting the SLM as more appropriate for addressing the spatial dependence in our data.

### 3.3 Results of SLM

The SLM yielded a statistically significant coefficient for the spatial lag term (*ρ* = 0.44, *p* = 0.00843), quantitatively confirming substantial spatial dependence between adjacent regions. Specifically, a one-unit increase in mumps incidence among neighboring areas was associated with a 0.44-unit increase in the target region’s incidence, demonstrating characteristic spatial spillover effects in infectious disease transmission.

After incorporating spatial spillover effects, all explanatory variables except years of education showed statistically significant associations (Table 2). Notably, compared with MLR, the SLM demonstrated several analytical advantages: First, the number of general practitioners - statistically insignificant in MLR - emerged as significant in SLM, highlighting the importance of accounting for spatial effects. Second, model fit indices showed marked improvement, with higher *R^2^* values, significantly increased *log-likelihood*, and reduced *AIC* values in SLM compared to MLR (Table 3).

**Table 3:** Goodness-of-fit of the four models.

Model selection criteria	MGWR	GWR	SLM	MLR
Moran’s I	−0.0476 (*p* = 0.9)	0.02 (*p* = 0.6177)	0.005 (*p* = 0.7359)	0.1703 (*p* = 0.00907)
R^2^	0.769	0.738	0.654	0.576
AIC	62.409	65.918	69.575	72.367
Log Likelihood	−21.278	−23.220	−27.787	−30.183

Diagnostic tests confirmed the SLM’s effectiveness, as residual spatial autocorrelation became non-significant, indicating successful capture of the underlying spatial dependence structure (Table 3). These results methodologically validate that the SLM effectively addresses specification bias caused by ignoring spatial autocorrelation in conventional regression analysis, thereby enhancing the reliability of research conclusions. The findings underscore the necessity of incorporating spatial dimensions when analyzing infectious disease patterns and determinants.

### 3.4 Results of local spatial regression models

#### 3.4.1 Model comparison and selection

This study successfully established both GWR and MGWR models. While the MGWR model showed slightly higher explanatory power (*R^2^* = 0.769 vs. 0.738), lower *AIC* value (62.409 vs. 65.918), and improved log-likelihood (−21.278 vs. -23.220) compared to GWR (Table 3), further examination of its bandwidth parameters revealed that nearly all explanatory variables operated at bandwidths approaching or exceeding 7,500 km. Given that the maximum inter-provincial distance in China is approximately 5,000 km, these bandwidths effectively encompass the entire study area, indicating that the MGWR model had largely degenerated into a global regression framework. Consequently, despite its marginally better goodness-of-fit, the MGWR model failed to provide meaningful local parameter estimates and thus did not offer genuine advantages in capturing spatial heterogeneity.

In contrast, the GWR model employed a fixed Gaussian kernel with an *AIC_c_*-selected bandwidth of 1,433,355.29 meters (approximately 1,433 km). At this bandwidth, each local regression included approximately 20–25 neighboring provinces, providing sufficient local observations for stable estimation while effectively capturing spatial heterogeneity. Both local spatial regression models substantially outperformed the conventional SLM (SLM: *R^2^* = 0.654, *AIC* = 69.575, *Log-likelihood* = −27.787), and residual spatial autocorrelation tests were statistically non-significant for both GWR (*Moran’s I* = 0.02, *p* = 0.6177) and MGWR (*Moran’s I* = −0.048, *p* = 0.9) (Table 3). Based on these comparative results, the study ultimately selected the GWR model as the primary analytical framework to investigate the spatially heterogeneous effects of various influencing factors on mumps incidence across Chinese provinces.

#### 3.4.2 Spatial heterogeneity of influencing factors

The detailed estimation results of the GWR model reveal that all five core explanatory variables significantly influenced mumps incidence, with notable spatial heterogeneity in both the direction and magnitude of their effects.

**Figure 5:**
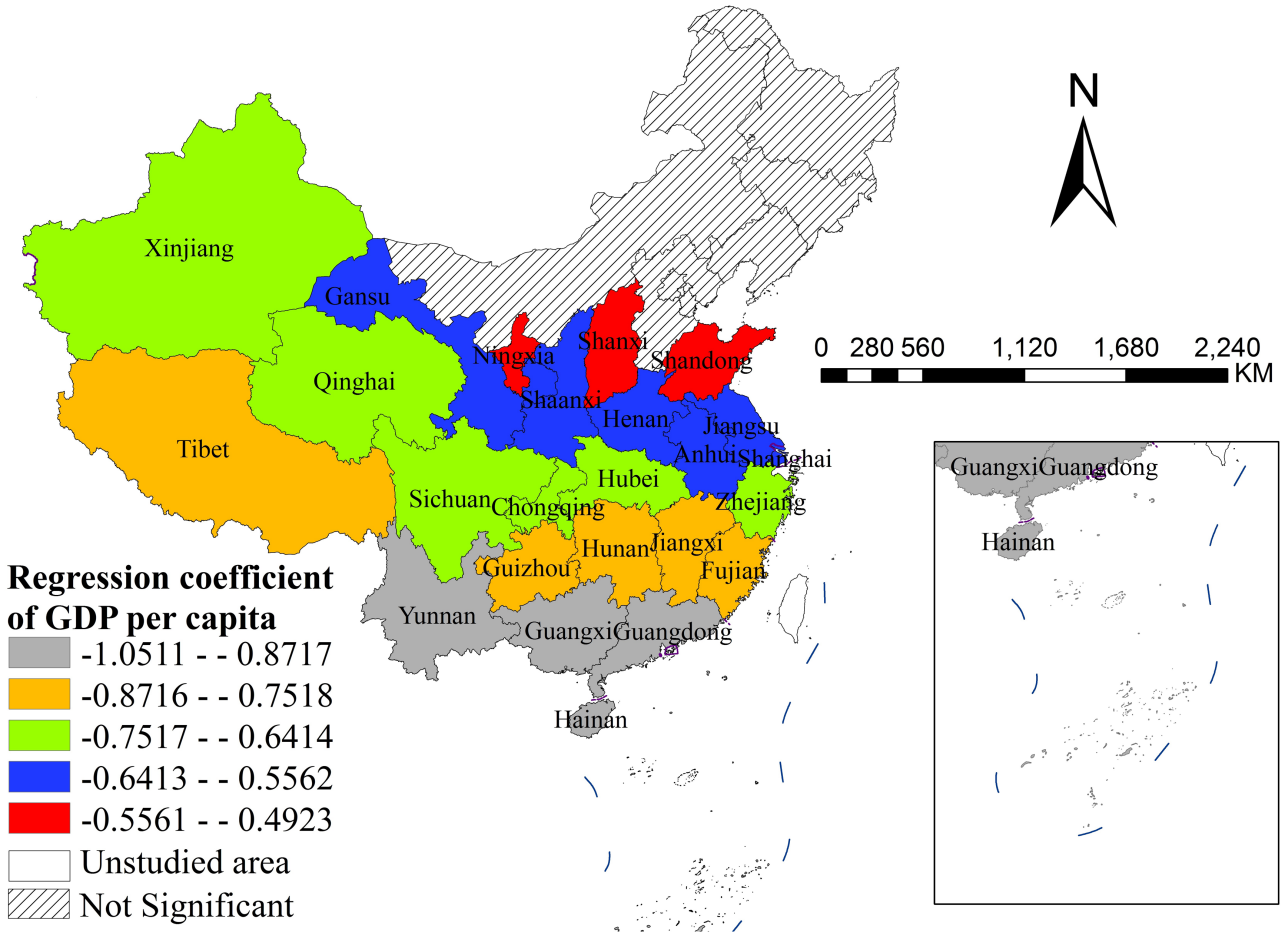
The spatial distribution of regression coefficient of GDP per capita.

Per capita GDP exhibited significant negative associations with disease incidence, with pronounced spatial heterogeneity in effect intensity across Chinese provinces. The strongest negative effects were concentrated in southern and southwestern provinces, namely Yunnan, Guangxi, Guangdong and Hainan, indicating that economic development exerts the most pronounced inhibitory impact on disease incidence in these regions (Figure 5). In contrast, the weakest significant negative effects were observed in northern provinces including Shandong, Shanxi and Ningxia. Most northeastern provinces (e.g., Heilongjiang, Jilin, Liaoning) and other northern regions showed non-significant associations, suggesting that per capita GDP is not a key influencing factor for disease incidence in these areas.

**Figure 6:**
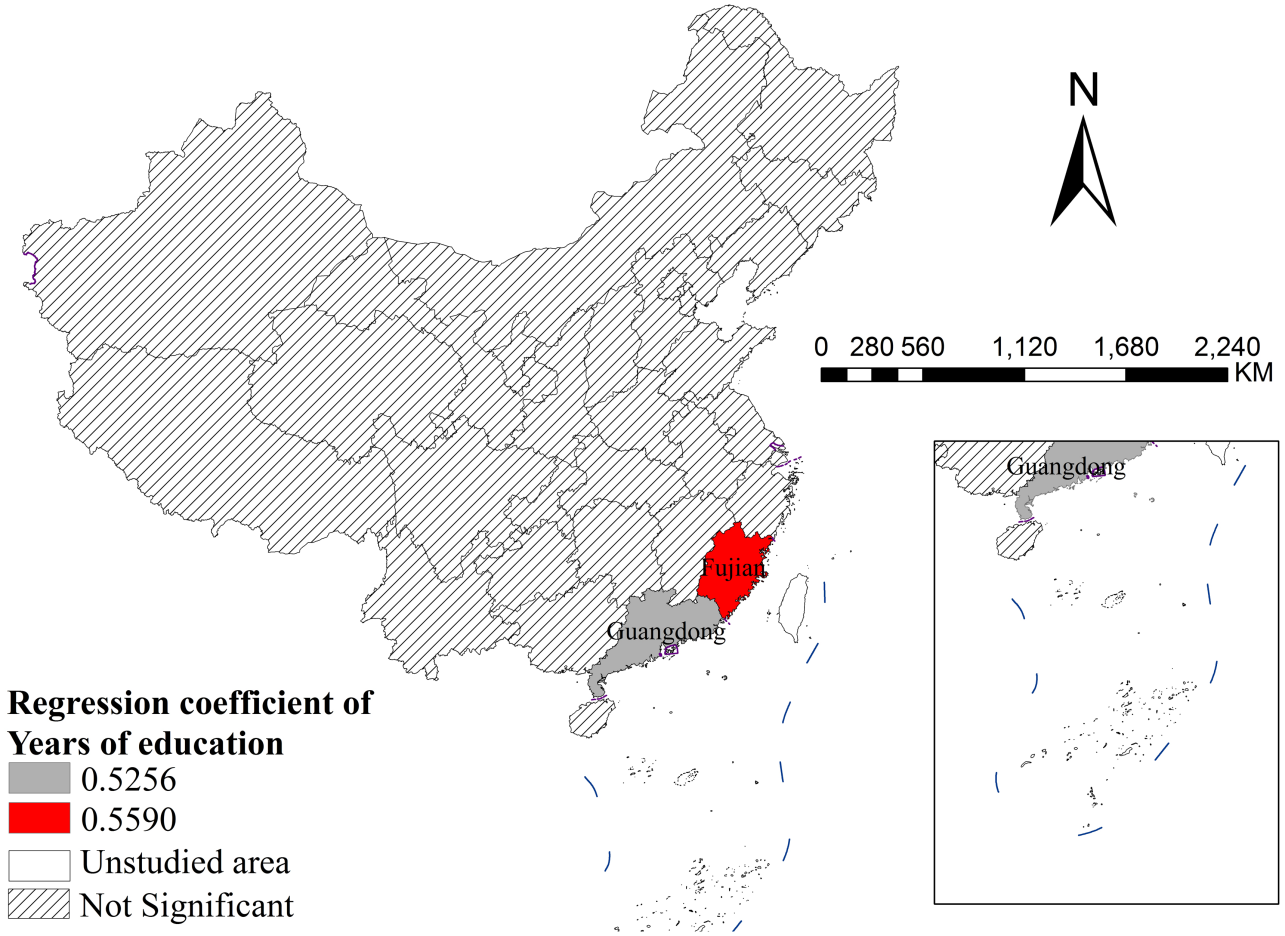
The spatial distribution of regression coefficient of years of education.

Average years of education showed significant positive associations with disease incidence only in Fujian (coefficient = 0.5590) and Guangdong (coefficient = 0.5256) in southern China. All other provinces across the country presented non-significant effects, suggesting that educational level has a limited and geographically concentrated impact on disease incidence in China (Figure 6).

**Figure 7:**
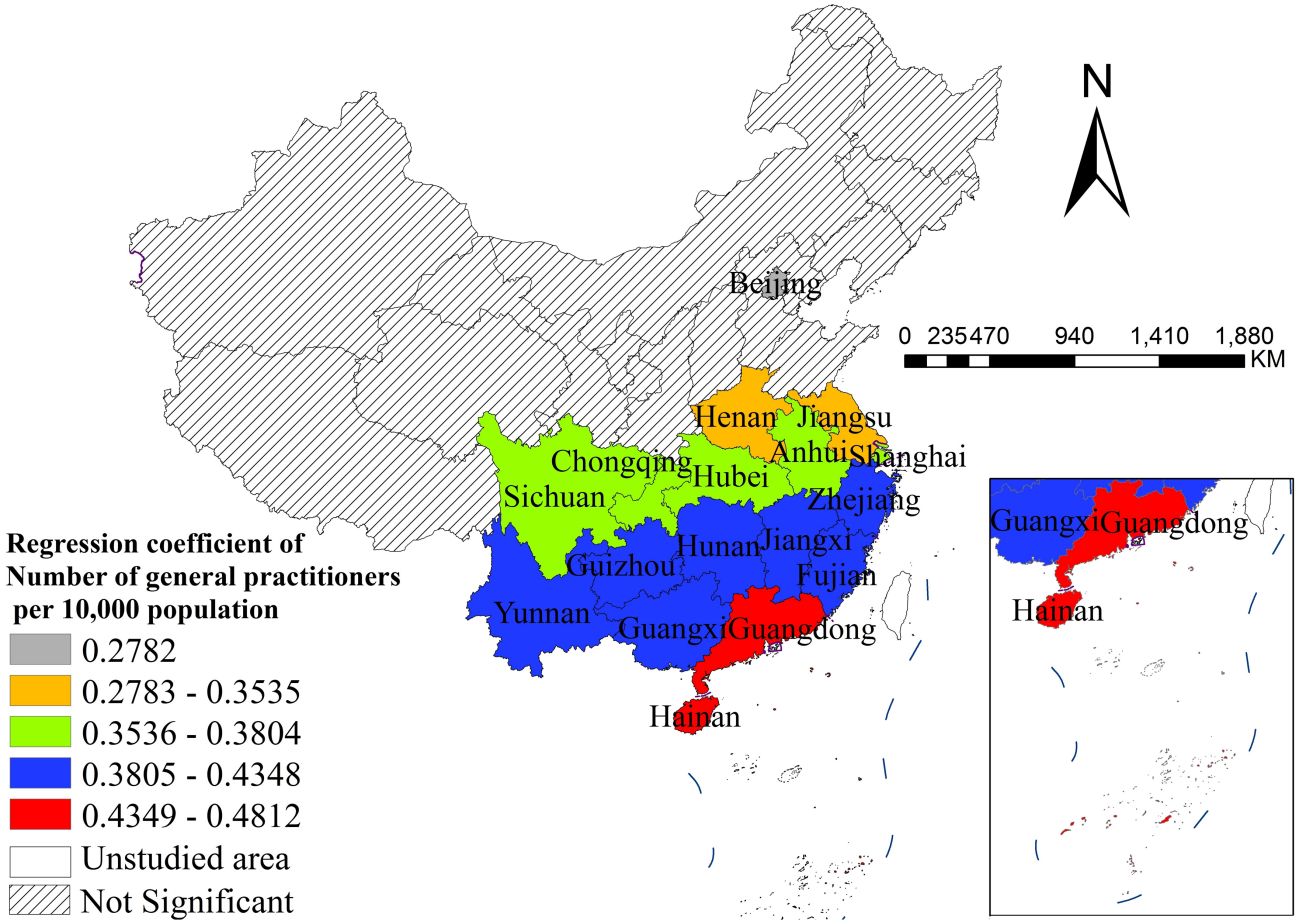
The spatial distribution of regression coefficient of number of general practitioners per 10,000 population.

**Figure 8:**
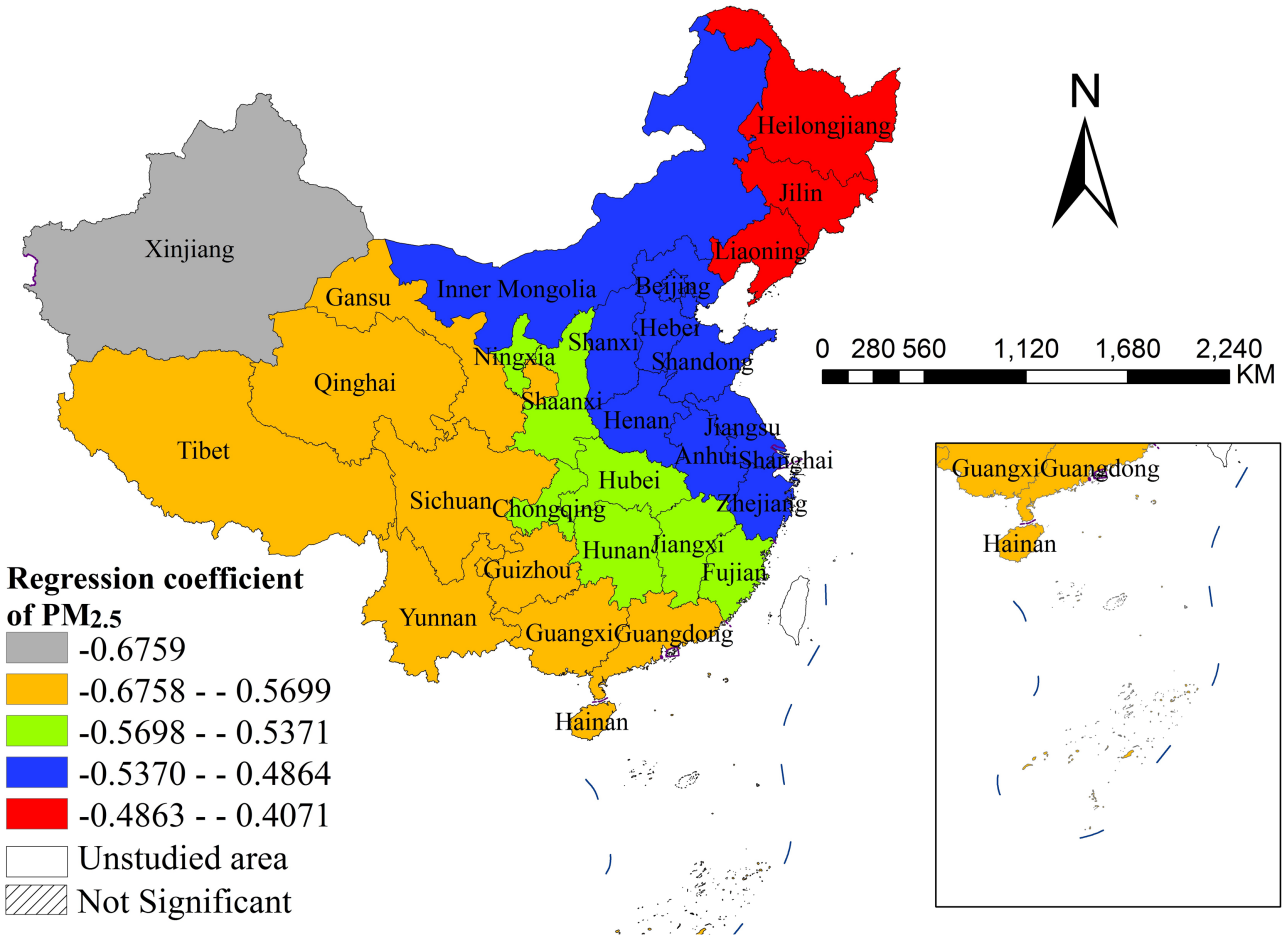
The spatial distribution of regression coefficient of PM_2.5_.

**Figure 9:**
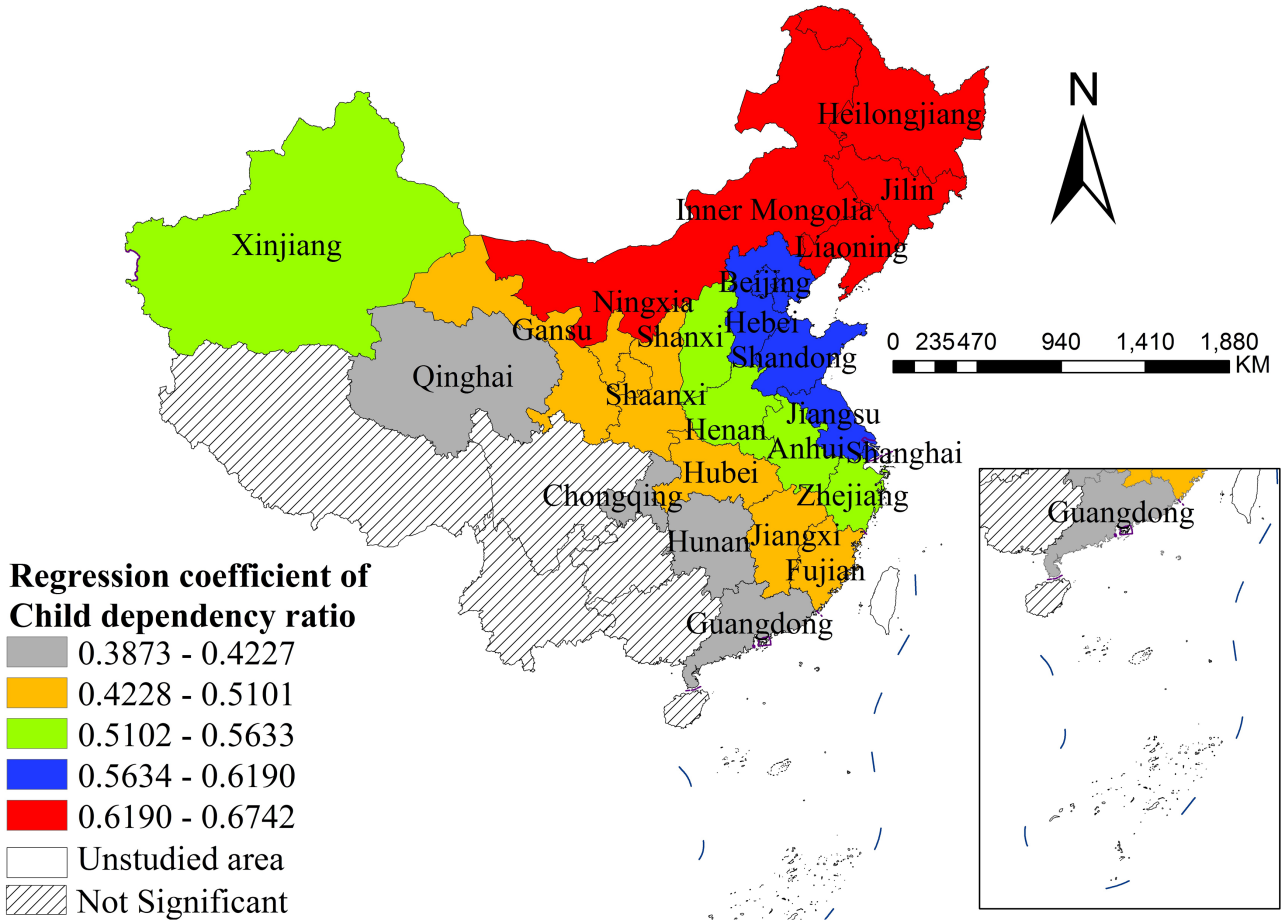
The spatial distribution of regression coefficient of child dependency ratio.

The density of general practitioners was significantly positively correlated with disease incidence, with distinct spatial heterogeneity. The strongest positive effects were observed in Guangdong and Hainan, while the weakest significant effects were concentrated in Beijing, Henan, and Jiangsu. All northeastern, northern and most western provinces showed non-significant associations, indicating that the supply of general practitioners has a geographically concentrated positive impact on disease incidence in southern and central China (Figure 7). PM_2.5_ concentration was significantly negatively correlated with disease incidence nationwide, with distinct spatial heterogeneity in effect size. The strongest negative effects were found in northwestern and southwestern China, while the weakest significant effects were concentrated in northeastern and eastern coastal provinces (Figure 8). The child dependency ratio was significantly positively correlated with disease incidence, with distinct spatial heterogeneity. The strongest positive effects were observed in northern and northeastern China, while the weakest significant effects were concentrated in southwestern and southern coastal provinces. Most southwestern provinces showed non-significant associations (Figure 9).

**Figure 10:**
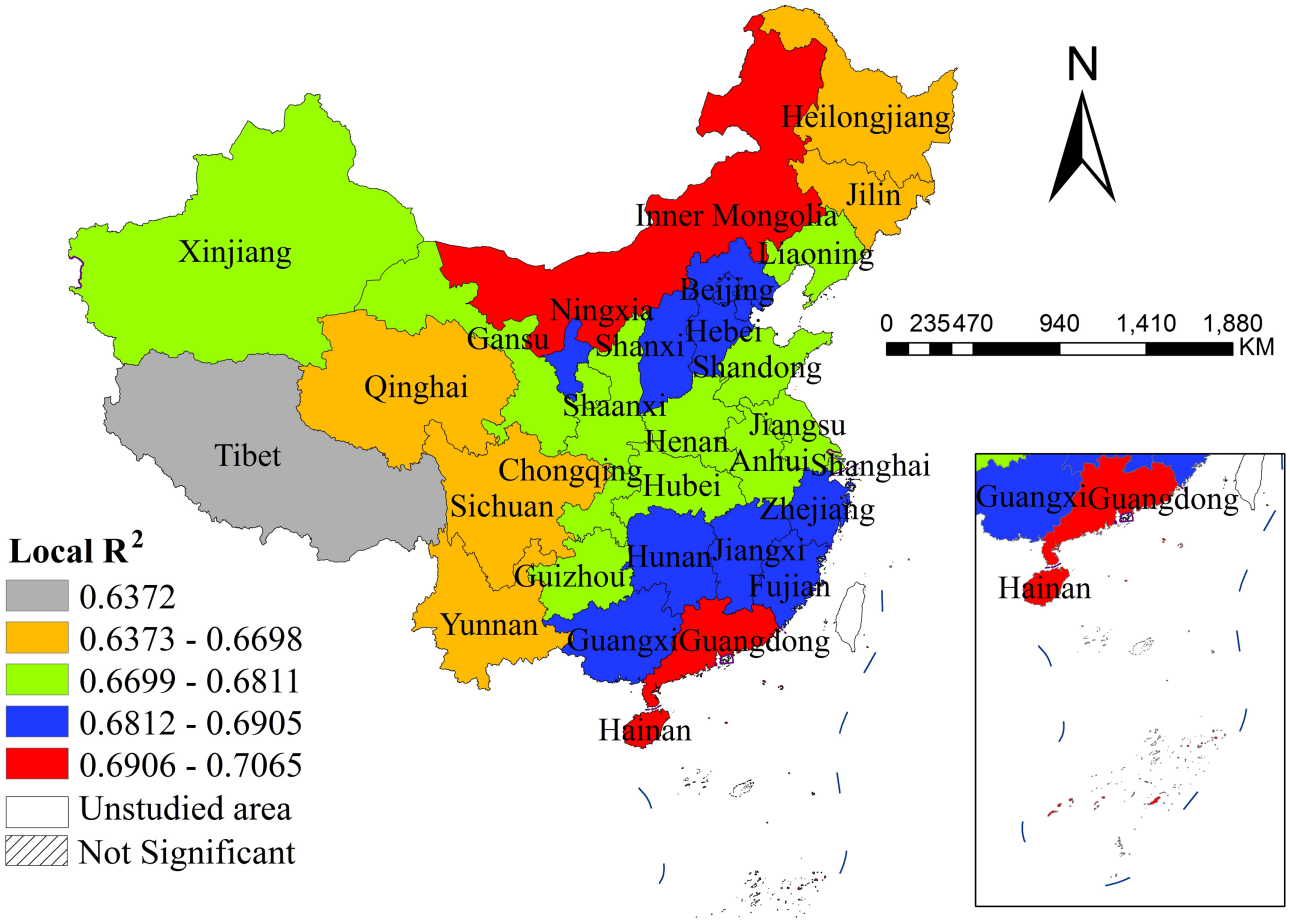
The spatial distribution of local R^2^.

The local *R^2^* values showed significant spatial heterogeneity in model explanatory power across China. The highest model fit was observed in Inner Mongolia, Guangdong and Hainan, while the lowest was found in Tibet. All provinces exhibited local *R^2^* values above 0.63, indicating that the selected variables consistently explained a large proportion of variance in disease incidence nationwide (Figure 10).

## 4 Discussion

By constructing a multi-model comparative analytical framework, this study systematically explored the spatial heterogeneity of mumps incidence in China in 2020 and its driving factors. The results showed that mumps incidence exhibited a west-high/east-low gradient distribution pattern with significant spatial autocorrelation. Unlike previous studies that predominantly employed global regression or single-scale local models, this study compared the performance of MLR, SLM, GWR, and MGWR within a unified framework, revealing the advantages and disadvantages of different models in capturing spatial heterogeneity.

Although MGWR was slightly superior to GWR in terms of goodness-of-fit, its optimal bandwidths for all variables exceeded 7,500 km, far beyond the maximum inter-provincial distance in China (approximately 5,000 km), effectively degenerating it into a global model. The fundamental reason for this phenomenon lies in the limited sample size (only 31 provincial-level administrative units): MGWR requires estimating bandwidths independently for each variable, and under small-sample conditions, the bandwidth optimization process tends to select larger bandwidths to reduce estimation variance, thereby causing the model to lose sensitivity to local heterogeneity. It should be noted that this is not an overfitting problem - MGWR had a lower *AIC* value and better overall fit, but the excessively large bandwidth prevented it from effectively revealing spatial heterogeneity. In contrast, GWR employs a unified optimal bandwidth (approximately 1,433 km), better balancing estimation stability and local sensitivity with a limited sample. Therefore, although MGWR has theoretical advantages, GWR may be a more robust choice in practical applications, particularly in studies with limited sample sizes.

From the perspective of spatial heterogeneity of influencing factors, the GWR model results showed that the negative effect of GDP per capita on mumps incidence was strongest in southwestern China, while non-significant in northeastern China. This finding suggests that the protective effect of economic development level on infectious disease prevention and control may have significant regional heterogeneity (28), potentially related to regional differences in healthcare resource allocation efficiency, vaccination coverage, and healthcare service accessibility. Years of education showed a significant positive association only in Fujian and Guangdong, which contradicts the hypothesis that education enhances health literacy and reduces disease risk. This may reflect that higher education levels in these regions are accompanied by higher population mobility density or more active socio-economic activities, thereby increasing transmission risk (1). Alternatively, this positive association may reflect reporting or detection bias, as Fujian and Guangdong have better healthcare infrastructure and more complete disease surveillance systems, leading to higher case ascertainment compared to less developed regions. This counterintuitive finding warrants further investigation in future research.

The positive effect of general practitioner density was most prominent in Guangdong and Hainan, while non-significant in northeastern and western regions. This may reflect that regions with higher general practitioner density also have stronger case detection and reporting capabilities, rather than a genuinely higher disease risk, suggesting the possible existence of “detection bias” or “reporting bias” (33). PM_2.5_ concentration exhibited a significant negative association nationwide, particularly in northwestern and southwestern China. This result is inconsistent with findings from some studies indicating that air pollution impairs respiratory mucosal immunity and increases infection risk (2, 34). It may reflect that highly polluted regions simultaneously adopt stricter public health measures (35) or population behavior changes (e.g., reduced outdoor activities, mask-wearing), thereby indirectly reducing mumps transmission opportunities. The positive effect of the child dependency ratio was most prominent in northeastern China, consistent with factors such as high child concentration, numerous group living settings, and uneven vaccination coverage in this region (1, 6).

Based on the above spatial heterogeneity characteristics, the following regionally differentiated prevention and control strategies are proposed: In high-incidence southwestern regions, priority should be given to improving vaccine coverage and cold chain capacity, increasing fiscal transfer payments. In northeastern regions, efforts should focus on strengthening morning inspections, absenteeism tracking, and emergency vaccination mechanisms in childcare facilities. In southeastern coastal regions, mobile population management and targeted health promotion should be strengthened, leveraging the health literacy advantages of highly educated populations to improve vaccination compliance. In northwestern regions, comprehensive respiratory protection interventions should be implemented in conjunction with high air pollution seasons (e.g., winter heating period), encouraging mask-wearing and ventilation.

This study has several important limitations. First, only 31 provincial-level administrative units were included in this study, which not only limits the ability to capture finer-scale spatial variations at municipal or county levels but also affects the robustness of MGWR due to the limited sample size, making it difficult to effectively reveal local spatial heterogeneity. Second, several key epidemiological variables were not included in the models due to data availability constraints, including vaccination coverage critical for mumps prevention, population mobility patterns, climate variables such as temperature and humidity, school density or contact patterns, and evolving prevention policies. The absence of these factors may affect the comprehensiveness of the findings. Third, although the cross-sectional design using data from a single year (2020) effectively identifies spatial patterns, it inherently lacks the capacity for robust causal inference. Finally, although 2020 represented the most recent year with available provincial-level mumps incidence data at the time of analysis, the concurrent COVID-19 pandemic may have introduced atypical patterns in disease transmission and reporting, potentially limiting the generalizability of our findings to non-pandemic periods.

Future research should address these limitations by incorporating higher-resolution spatial data, expanding variable coverage, and adopting longitudinal designs. Additionally, while the spatial regression models employed here effectively capture heterogeneity, future studies could benefit from integrating artificial intelligence algorithms (e.g., random forests, neural networks) to capture complex nonlinear relationships and improve predictive performance.

## 5 Conclusion

This study demonstrates that the drivers of mumps incidence in China vary significantly across regions. Methodologically, while MGWR offers theoretical advantages, its effectiveness is limited by small sample sizes. GWR proved more robust for capturing spatial heterogeneity with 31 provincial units. Substantively, GDP per capita, education, general practitioner density, PM_2.5_, and child dependency ratio all exhibited spatially varying effects, challenging one-size-fits-all prevention strategies. These findings provide a scientific basis for regionally differentiated mumps control and offer methodological guidance for spatial epidemiological studies with similar data constraints.

## Data Availability

The original contributions presented in the study are included in the article/Supplementary material, further inquiries can be directed to the corresponding authors.

## Footnotes

1
https://www.phsciencedata.cn/Share/
